# Induction of remission in autoimmune polyglandular syndrome type three (APS III): An old drug with new perspectives

**DOI:** 10.1002/ccr3.1827

**Published:** 2018-10-02

**Authors:** Magdy Mohamed Allam, Hanaa Tarek Hussein Elzawawy

**Affiliations:** ^1^ Internal Medicine Department Alexandria University Student Hospital (AUSH) Alexandria Egypt; ^2^ Internal Medicine Department, Faculty of Medicine Alexandria University Alexandria Egypt

**Keywords:** autoimmune polyglandular syndrome, autoimmune polyglandular syndrome type III, azathioprine, Grave’s disease, Hashimoto’s thyroiditis, hyperthyroidism, hypothyroidism, type 1 diabetes mellitus, vitiligo

## Abstract

The autoimmune polyglandular syndrome is a sequential chain of autoimmune events. Whenever diagnosed, the clinician's target should be induction of remission and if possible hindering its progression especially if associated with refractory vitiligo, resistant Grave's, or unexplained hyperglycemia in T1DM. Azathioprine could be used for induction of remission in autoimmune polyglandular syndrome type three especially with vitiligo and autoimmune thyroiditis.

## 1 BACKGROUND

The main characteristics of autoimmune polyglandular syndrome type three (APS III) are autoimmune thyroid diseases associated with other autoimmune diseases, excluding Addison's disease and hypoparathyroidism. We reported two cases of APS III. The first case started by refractory vitiligo followed by autoimmune hepatitis (AIH) and Hashimoto's thyroiditis with mild hypothyroidism. The second case started with Type 1 diabetes mellitus (T1DM) followed by resistant Grave's disease then AIH developed. When azathioprine was administered as a maintenance therapy in AIH, the thyroid antibodies were reduced to low levels with remission of vitiligo and normalization of thyroid functions in association with decreased insulin requirements in T1DM. APS III should be treated as a sequential chain of autoimmune events, and remission could be induced by azathioprine administration mainly in refractory vitiligo and autoimmune thyroiditis.

Autoimmune polyglandular syndromes (APS) include a diverse group of clinical conditions characterized by dysfunction of multiple endocrine glands due to loss of immune tolerance. In 1980, Neufeld and Blizzard set out the first APS classification, dividing this syndrome into three subtypes.^1^ Recently, APS are classified into four main types by Betterle et al; *APS type I*, occurs in childhood, has been defined as a combination of mucocutaneous candidiasis, hypoparathyroidism, and adrenal insufficiency (at least two must be present). APS type II or Schmidt syndrome is more common in the adult population and is characterized by primary adrenal insufficiency (always present) with autoimmune thyroid disease, and/or diabetes mellitus type I.^2^

*APS type III* includes autoimmune thyroid disease in association with other autoimmune diseases other than Addison's disease and hypoparathyroidism. C. Betterle et al subdivided APS III into four groups, according to diseases accompanied autoimmune thyroid diseases: APS III‐A contains all endocrine diseases, APS III‐B encloses gastrointestinal lesions & pernicious anemia, APS III‐C includes skin lesions, myasthenia gravis, and the nervous system), and finally, APS III‐D comprises all collagen diseases and vasculitis. APS type IV contains combinations that do not be included in the previously reported groups.^2^

For the optimal management of autoimmune diseases, three sequential steps are needed: first, induction of remission or preventing disease progression; second, achieving complete remission without drug dependence; and finally, maintain remission without relapses. However, the treatment goal of APS does not follow these steps and varies according to the characteristics of the disease, aiming at controlling the condition or hormonal replacement therapy for glands insufficiency.

Azathioprine (Aza), a synthetic purine analog derived from 6‐mercaptopurine, acts by disrupting the nucleic acid synthesis and interfering with T‐cell activation (the main cell involved in the pathogenesis of APS). The most recognized uses of Aza are in the treatment of rheumatoid arthritis and prevention of rejection in renal transplantation. Also, it may be used in the treatment of other autoimmune disease and some malignancies.

We report how azathioprine helps in the induction of remission and even reversal of some components in two different cases of APS III.

## 2 CASE PRESENTATION

### 2.1 CASE one

A 22‐year‐old female was admitted to the department of internal medicine because of fatigue, anorexia, upper abdominal pain, and mild jaundice. In addition to a fourfold elevation in aminotransferase levels, anti‐smooth muscle antibody (ASMA) and antinuclear antibody (ANA) were also elevated (1/240, and 1/320, respectively). Abdominal ultrasound was done which showed mild enlargement of the liver with a homogenous pattern. The diagnosis of autoimmune hepatitis (AIH) was established after a liver biopsy. The patient had a history of vitiligo, which firstly appeared in the circumoral area at the age of 17 then spread to the feet, hands, scalp, inguina, and breasts. She started treatment of generalized vitiligo vulgaris with narrow‐band ultraviolet B (UVB) radiation three times weekly with a topical corticosteroid (CS) for 6 months, yet only mild improvement has occurred. After that, she was maintained on oral CS and tacrolimus ointment (0.1%) with oral cholecalciferol (due to vitamin D deficiency; 25‐hydroxyvitamin *D* = 15 ng/mL) for three months without satisfactory results. One year later, a vitiliginous patch started to appear at the hair roots in the forehead, so she started to get intralesional scalp triamcinolone 0.1% every week for 5 months without significant improvement. Autologous transplantation of melanocytes using suction blister method was done which was followed by incomplete repigmentation of the transplanted area. Eventually, the patient became depressed and relied only on cosmetic concealer techniques with vitamin D supplementation. The vitiligo became static in its course, and the patient refused to be engaged in any further therapeutic procedures. The patient also has a past history of allergic rhinitis since childhood, and her both parents had Hashimoto's thyroiditis.

On examination; the patient's height: 170 cm, weight: 67 kg, body mass index (BMI): 23.2 kg/m^2^, BP:90/70 mm Hg and pulse: 78 minutes. The patient was presented with a generalized form of vitiligo, based on numerous amelanotic patches on the hands, forearms, feet, face, and trunk. Mild jaundice and right hypochondrial tenderness were also detected.

Autoimmune and hormonal profiles were assessed. Because of mildly elevated TSH and anti‐TPO (anti‐thyroid peroxidase antibody) with normal free T4 and free T3 levels, Hashimoto's thyroiditis with subclinical hypothyroidism was diagnosed. Ultrasound neck revealed increased thyroid gland volume with heterogeneous hypoechoic pattern confirming the diagnosis. So, treatment with L‐thyroxin 25 μg/d was sufficient to normalize the thyroid function.

Furthermore, complete blood picture showed mild leukopenia, macrocytic anemia, and thrombocytopenia [(Erythrocyte count (3.2 × 106/μL), hemoglobin (11.3 g/dL), MCV (109 fL), MCHC (35 g/dL), platelet count (132 × 10^3^/μL), and leukocyte count (3.7 × 10^3^/μL)]. The suspicion of pernicious anemia was raised. High titers of gastric parietal cells antibodies (APCA) and low vitamin B12 (3.7, normal range 145‐914 pg/mL) confirmed the diagnosis. An upper gastrointestinal (GI) endoscopy was performed; the macroscopic and histological examination revealed the presence of atrophic gastritis. These data confirmed the presence of autoimmune gastritis with pernicious anemia.

Adrenal insufficiency and hypogonadism were excluded. Other autoantibodies were negative including islet‐cells antibodies (ICA), glutamic acid decarboxylase autoantibodies (GAD Abs), and double‐stranded DNA antibodies (Anti‐ds DNA Ab) were negative. Based on the constellation of Hashimoto's thyroiditis, autoimmune gastritis, AIH, and vitiligo, APS III type (B + C) was diagnosed. The patient was discharged from the hospital on prednisolone 60 mg, 25 μg/d L‐thyroxin, and injectable B12 replacement therapy. Liver enzymes were performed weekly which started to decline with improvement of the patient general condition.

**Figure 1:**
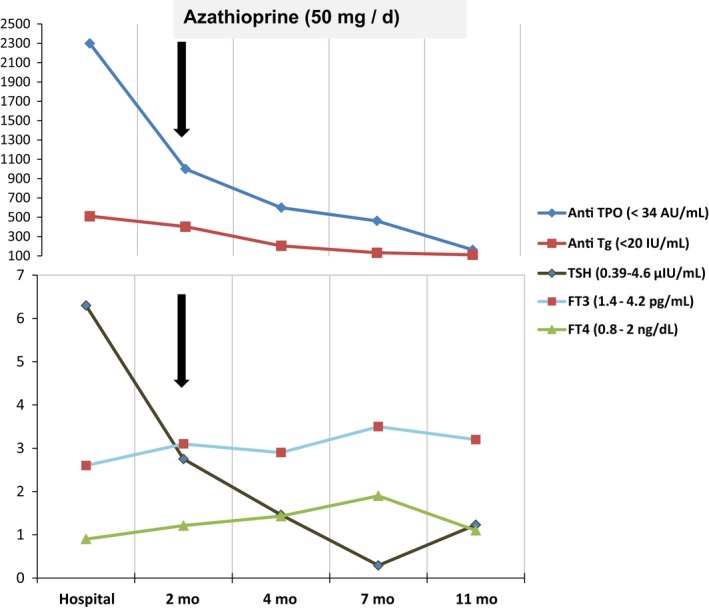
Case one. The effect of azathioprine on the course of Hashimoto's thyroiditis. Anti‐TPO, Anti‐thyroid peroxidase antibody; Anti Tg, Anti‐thyroglobulin antibody; TSH, Thyrotropin; FT3, Free triiodothyronine; FT4, free thyroxine; mo, months

The prednisolone was tapered over 4 weeks, and Aza 50 mg was introduced daily after achieving normal liver enzymes and improvement in the general condition of the patient. Two months later, surprising repigmentation of the feet and forearm vitiliginous patches were started. On the following visit after two months, the patient suffered from palpitation in concordance with decreased levels of TSH and anti‐TPO. So, we started to decrease the dose of levothyroxine till stoppage after 3 month (Figure 1).

**Figure 2:**
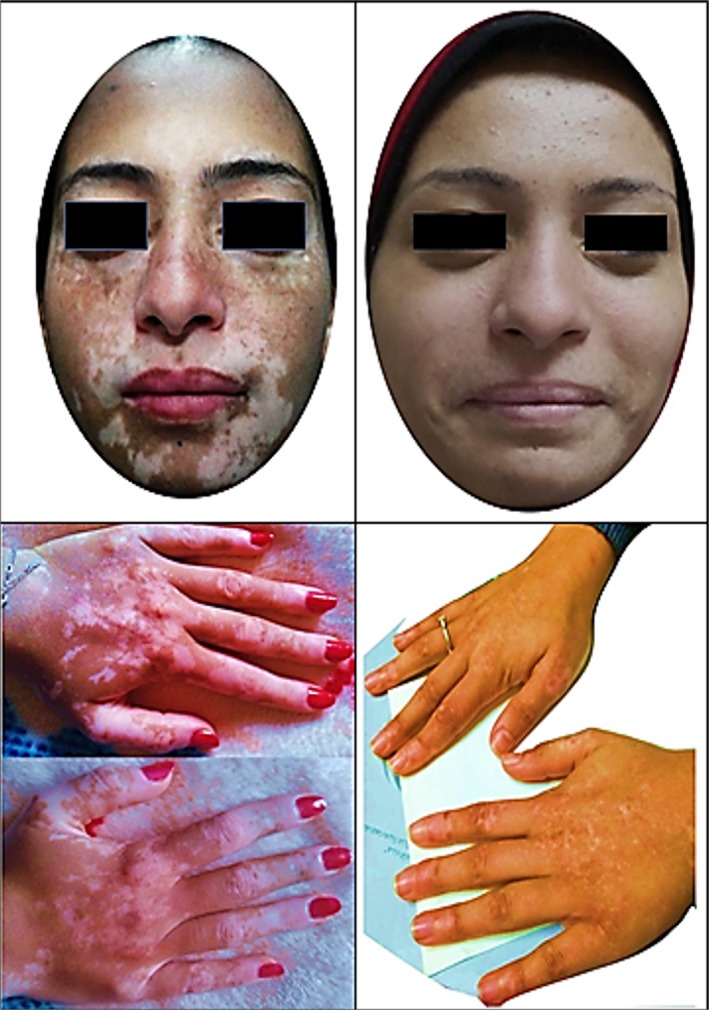
Case one. The clinical course of vitiligo vulgaris before (left), and 12 mo after azathioprine intake (right)

During the following year, the only reported side effect was mild leukopenia (leukocyte count = 3.5 × 10^3^/μL) which did not need a further intervention after hematological consultation. Now, she is maintained on Aza 25 mg daily and cholecalciferol 50 000 IU monthly. The patient is feeling quite fine, and more than 95% of vitiligo has improved together with remission of thyroid dysfunction (Figure 2).

### 2.2 CASE two

A 28‐year‐old man was admitted to the department of internal medicine because of fatigue, thirst, frequency, five‐kilogram weight loss, diarrhea, and palpitation. The patient was diagnosed as type 1 diabetes mellitus (T1DM) at the age of 19 and was treated with intensive basal‐bolus insulin therapy (70 units per day). He used to smoke around twenty cigarettes per day for ten years. The patient had no family history of APS, autoimmune thyroid disease or any other immunological disorders. On examination; his height: 157 cm, weight: 59 kg, body mass index (BMI): 23.9 kg/m^2^, BP: 100/70 mm Hg, pulse: 130 beat/min. Neurological examination revealed signs of distal symmetric polyneuropathy. His thyroid gland was just palpable without ophthalmopathy. Because of high blood glucose (350 mg/dL), acetone in urine, and metabolic acidosis, he was diagnosed as diabetic ketoacidosis (DKA).

Since low TSH, high free T4 (FT4), high free T3 (FT3), positive thyroid stimulating hormone receptor antibody (TRAb), and a diffuse homogenous thyroid gland enlargement with increased blood flow by thyroid ultrasound, Grave`s disease was diagnosed. After exclusion of all precipitating causes of DKA and confirming patient's adherence to treatment and diet, Grave`s disease (hyperthyroid state) was found to be the only precipitating cause of DKA. IV fluids, insulin infusion, Lugol`s iodine 50 mg with 30 mg carbimazole and propranolol 120 mg/d were started till the control of thyroid functions, and blood glucose was achieved after 10 days. The patient has discharged on insulin glargine 30 units, Aspart 25 units, 30 mg carbimazole, and propranolol 120 mg/d.

ICA, GAD, ASMA (1/240), and ANA Abs (1/160) were positive. Serum vitamin B12 (902 pg/mL) and complete blood count test were normal. Anti‐ds DNA Ab and tissue transglutaminase antibodies were negative. Also, APCA, hepatitis B surface antigen (HBsAg) and hepatitis C virus antibody (HCVAb) were negative. Adrenal insufficiency and hypogonadism were excluded.

During the follow‐up visits, the blood glucose was poorly controlled, and thyroid hormones were persistently high despite patient's adherence to treatment. The patient refused to proceed with surgery or radioactive iodine options. So, the dose of carbimazole was increased up to 90 mg/d, and propranolol to 240 mg/d followed by switching to propylthiouracil (PTU) at a dose of 900 mg/d. The patient's symptoms became controlled with high normal thyroid hormones. Normal liver functions and complete blood count test were assured every visit.

Six months later, the patient was hospitalized, owing to mild jaundice, and severe anorexia. Laboratory tests showed elevated levels of aminotransferases (AST = 277 IU/L, ALT = 313 IU/L) and total bilirubin (1.6 mg/dL). The most prevalent differential diagnoses included autoimmune hepatitis, PTU induced hepatitis, hepatitis viral infection, and Grave's disease. Since the patient had positive ANA, ASMA, and liver biopsy, and negative viral markers, the diagnosis of autoimmune hepatitis was settled. Based on the combination of Grave's disease, T1DM, and AIH, the diagnosis of APS III type (A + B) was established.

So, we started prednisolone 60 mg per day for one month accompanied with daily Aza 50 mg, and the blood glucose was closely monitored and controlled using insulin glargine 60 units,and Aspart 65 units per day. PTU was stopped with the introduction of 50 mg Lugol`s iodine. After remission of liver enzymes, PTU was reintroduced in stepping up doses in association with stepped down prednisolone till stoppage over 3 months then the patient was maintained daily on 50 mg Aza and 300 mg PTU.

**Figure 3:**
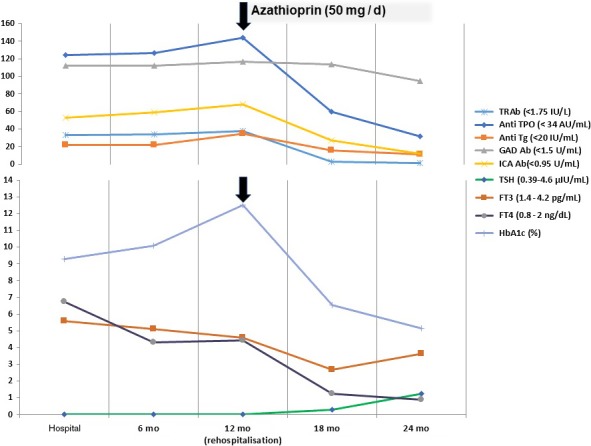
Case two. The effect of azathioprine on the laboratory course of Grave's disease and T1DM. Anti‐TPO, Anti‐thyroid peroxidase antibody; Anti Tg, Anti‐thyroglobulin antibody; TSH, Thyrotropin; FT3, Free triiodothyronine; FT4, free thyroxine; ICA, islet‐cells antibodies; GADAb, glutamic acid decarboxylase autoantibodies; mo,months

During a year following Aza initiation, no side effects were reported, and the patient started to decrease the insulin dosage mutually with decreasing ICA Ab titer. Also, the thyroid function was controlled rapidly coinciding with the decline of thyroid antibodies to almost undetectable levels. Now, the patient is maintained on Aza 50 mg daily, insulin glargine 25 units, insulin Aspart 25 units, and anti‐thyroid drugs were stopped (Figure 3).

## 3 DISCUSSION

### 3.1 Autoimmune polyglandular syndrome type three (APS III)

It is an adult type of APS characterized by the association of endocrine and non‐endocrine organ‐specific autoimmune disorders. The presence of autoimmune thyroiditis and the absence of adrenal insufficiency are crucial for the diagnosis of all subtypes of APS III. The exact prevalence of APS is unknown, although APS III accounts for 41.5% of all APS.^3^ The diagnosis is made after the presence of two components, yet it may be delayed for 29 years till the onset of consecutive autoimmune diseases.^3^ The most frequent non‐endocrine diseases associated with APS III are vitiligo, atrophic gastritis/pernicious anemia, and collagen diseases (7%, 9.8%, and 5% respectively).^4^ Autoimmune hepatitis was rarely recognized in APS.^5^

*Type 1DM* is characterized by the presence of insulitis and β‐cell autoantibodies resulting in an autoimmune destruction of insulin‐producing β cells.^6^ As seen in the second case, thyroid dysfunction was at the top of the differential diagnosis list of DKA in T1DM patients after exclusion of the usual precipitating factors. Hyperthyroidism increases glucose resorption and hepatic glucose release leading to hyperglycemia. Also, it leads to insulin resistance and an increased release of fatty acids causing ketoacidosis.^7^ Moreover, in 60% of APS type III patients, Grave's hyperthyroidism may occur prior to T1DM in a mean time of seven years. Zinc transporter 8 autoantibodies (ZnT8As), and glutamic acid decarboxylase autoantibodies (anti GADAbs) are frequently present in APS type III than in isolated T1D.^8^

*Autoimmune thyroiditis* was present in both cases. The hallmarks of Hashimoto's thyroiditis are the sonographic hypoechoic pattern and the high rate of positivity of thyroglobulin and thyroperoxidase antibodies (anti‐TPO). The 20 years follow‐up study of the Whickham cohort demonstrated that high anti‐TPO mainly and high TSH (especially >5.0 μU/mL) are predictive of thyroid failure with an annual incidence of 4.3% which is raised to 50% in TPO‐antibody positive T1DM patients.^9^ High anti‐TPO level per se has many adverse health impacts, from increased coronary heart disease risk factors^10^ to poor pregnancy outcomes and even neuropsychiatric disorders in euthyroid patients.^11^ On the other hand, anti‐TSH receptor antibody (TRAb) levels may have a role as a promoter of Grave's ophthalmopathy and a crucial effect in the development of fetal hyperthyroidism throughout pregnancy.^12^

*Vitiligo* results from loss of functional melanocytes (pigment‐producing cells). It is characterised by the appearance of white patches on the skin and sometimes accompanied by whitening of the hair. The most common form of vitiligo is symmetrical nonsegmental vitiligo or vitiligo vulgaris. As in case one, the main theory explained the integration of vitiligo in the APS that anti‐thyroid hormone antibodies may cross‐react with tyrosinase or acetylcholinesterase antibodies causing impairment and loss of melanin production in the skin. So thyroid antibodies are recommended to be measured in patients with vitiligo.^13^ The standard therapy of non‐segmental vitiligo is the avoidance of triggering/aggravating factors, and NB‐UVB therapy may be in combination with systemic/topical therapies. The treatment of refractory vitiligo due to immune‐mediated mechanism, as in case one, is an important issue as treatment is not specified; it needs a long period of time with unsatisfactory results and high psychological burden.^14^

### 3.2 Management of Autoimmune polyglandular syndrome type three

Guidelines for the management of APS recommend serologic and functional screening for associated autoimmune diseases in patients with a monoglandular autoimmune disease at diagnosis and during follow‐up at least every 2 years. Screening should be performed to detect other endocrine diseases before the development of potentially severe acute complications (eg adrenal crisis or ketoacidosis) or chronic complications (eg mothers with TRAb inducing neonatal thyrotoxicosis, microvascular complications, and early retention of residual pancreatic beta‐cell function in T1DM).^15^ Till now the treatment of APS is organ‐specific therapies aiming at replacing the hormonal deficiency of an already destructed glands or preventing the development of complications of subclinically affected organs.

However, the components of APS could be predicted during long subclinical prodromal phases that express a progressive glandular destruction. This is mediated by a chronic inflammatory infiltrate of T‐cell lymphocytes (mainly), and production of organ‐specific autoantibodies before overt disease develops. Considering this, APS should be treated as sequential chains of autoimmune events could be prevented or hindering its progression.

So, attempts have been made to intervene during this prodromal phase, especially in T1DM prevention as a single disease, but the results were disappointing regarding the efficacy and side effects. Aza has not been tested for APS remission before as a whole entity, but it gave controversial results in selected components of APS.^16^

### 3.3 Azathioprine in management of Autoimmune polyglandular syndrome type three

Aza induces apoptosis of T cells, the main cells involved in the pathogenesis of APS, decrementing the number of circulating NK‐cells, neutrophils, and monocytes. Aza has a relatively slow onset of action around 6‐8 weeks.^17^ Regular monitoring of peripheral blood counts, kidney, and liver function tests should be done monthly. The most common side effect of Aza is gastrointestinal disturbance which decreases by dose adjustment or taking the medication with food. It can cause bone marrow suppression and hepatotoxicity mainly in a patient with a low thiopurine methyltransferase (TPMT) and xanthine oxidase (XO) enzymes. This side effect has been reduced by screening for TPMT, which regulates a key step in Aza metabolism while decreased XO activity is frequently mediated by medications such as allopurinol. Hence, Aza has safety, tolerability, wide therapeutic index, and cost advantages over immunosuppressants.^18^

Aza has been tested as a single agent for inducing remission in T1DM, but it failed to induce remission in recent‐onset T1DM patients. However, in combination with prednisone, Aza has been associated with increased remission rates in T1DM as indicated by lower insulin requirements and improved C‐peptide levels, yet remission could not be sustained beyond 1 or 2 years and is associated with considerable side effects mainly due to prednisone.^19^, ^20^ Only one study showed that triple therapy of Aza in combination with prednisolone plus ultraviolet radiation in vitiligo was more likely to achieve 75% of skin repigmentation 4 months after treatment.^21^

## 4 Conclusion

An autoimmune polyglandular syndrome should be treated as a sequential chain of autoimmune events. The development of other autoimmune disease may be suspected with unexplained loss of control or treatment refractoriness of the overt component. Hence, the aim of management is an induction of remission and if possible hindering its progression. Azathioprine could induce remission of APS III sequential events (mainly in refractory vitiligo and resistant autoimmune thyroiditis). A randomized controlled trial will be needed to prove this.

## INFORMED CONSENT

Written informed consent was obtained from the patient for publication of this case report.

## CONFLICT OF INTEREST

The author states that there are no conflicts of interest.

## AUTHORSHIP

MA: participated in patient management and data collection, contributed to the interpretation of the case, and critically reviewed the manuscript. HE: participated in data collection, contributed to the interpretation of the case, and critically reviewed the manuscript. All authors were major contributors. All the authors of this paper have reviewed the document in its entirety and are in agreement with the structure and content.
